# Effect of PCSK9 Inhibition on the Plasma Proteome: A SPIRE SubStudy

**DOI:** 10.1161/ATVBAHA.123.319272

**Published:** 2023-06-15

**Authors:** Jordan M. Kraaijenhof, Tjerk S.J. Opstal, Jan H. Cornel, Dipender Gill, G. Kees Hovingh, Alberico L. Catapano, Wolfgang Koenig, Paul M Ridker, Erik S.G. Stroes, Nick S. Nurmohamed

**Affiliations:** Department of Vascular Medicine, Amsterdam University Medical Centers, University of Amsterdam, The Netherlands (J.M.K., G.K.H., E.S.G.S., N.S.N.).; Department of Cardiology, Radboud University Medical Centre, Nijmegen, the Netherlands (T.S.J.O., J.H.C.).; Department of Cardiology, Northwest Clinics, Alkmaar, the Netherlands (T.S.J.O., J.H.C.).; Department of Epidemiology and Biostatistics, School of Public Health, Imperial College London, United Kingdom (D.G.).; Department of Pharmacological and Biomolecular Sciences, University of Milan, Milano, Italy (A.L.C.).; IRCCS Multimedica, Milano, Italy (A.L.C.).; Deutsches Herzzentrum München, Technische Universität München, Munich, Germany (W.K.).; German Centre for Cardiovascular Research (DZHK e.V.), partner site Munich Heart Alliance, Germany (W.K).; Institute of Epidemiology and Medical Biometry, University of Ulm, Germany (W.K.).; Divisions of Preventive Medicine and Cardiovascular Diseases, Brigham and Women’s Hospital, Harvard Medical School, Boston, MA (P.M.R.).; Department of Cardiology, Amsterdam University Medical Centers, Vrije Universiteit Amsterdam, The Netherlands (N.S.N.).

**Keywords:** cardiovascular disease, inflammation, lipid-lowering therapy, phenotype, proteomics

Individuals treated with contemporary secondary prevention therapies remain at considerable risk for atherosclerotic cardiovascular disease events. Many novel therapeutic strategies to lower residual atherosclerotic cardiovascular disease risk are being introduced, but the cost-benefit and specificity of these therapies necessitate reliable identification of patients that stand to gain adequate benefit. The plasma proteome has emerged as an intermediate phenotype for atherosclerotic cardiovascular disease, enabling personalized treatment choices based on individual high-risk protein signatures. However, the clinical benefit of the use of these high-risk signatures as tools to guide-specific interventions remains to be established. In a substudy of the LoDoCo2 (Low-Dose Colchicine for Secondary Prevention of Cardiovascular Disease study), 1 month of colchicine treatment resulted in marked changes in circulating levels of 60 proteins, supporting its broad anti-inflammatory effects and offering potential to identify the best treatment responders.^1^ For selective low-density lipoprotein cholesterol (LDL-C) lowering by PCSK9 (proprotein convertase subtilisin/kexin type 9) inhibition, multiple studies have suggested that its potent LDL-C lowering effect coincides with marked reductions in both cellular and arterial plaque inflammation, despite unaltered hs-CRP (high-sensitivity C-reactive protein) levels.^2–4^ As previously reported from SPIRE (Studies of PCSK9 Inhibition and the Reduction of Vascular Events), PCSK9 inhibition with bococizumab given as an adjunct to statin therapy additionally reduced plasma LDL-C (median change −65.4%) with no impact on hs-CRP levels (median change 0.0%).^4^ In the present study, we set out to investigate potential anti-inflammatory effects of PCSK9 inhibition by performing serial plasma proteomic profiling in a subset of the SPIRE-1 and 2.

In 190 patients participating in the SPIRE-1/2 trials (NCT01975376; NCT01975389; all provided written informed consent for the use of plasma samples), 184 targeted proteins were measured semi-quantitatively at baseline and after 12 months of bococizumab treatment using Cardiovascular II and III panels (Olink, Proximity Extension Assay, Uppsala, Sweden). Anonymized proteomics data has been made available (DOI: 10.7910/DVN/8T8XJJ). Ninety-six patients received placebo and 94 received 150 mg of bococizumab, once every 2 weeks. Considering the development of auto-antibodies in some patients,^5^ bococizumab patients with <15% LDL-C reduction after 12 months or missing follow-up LDL-C values were excluded, resulting in a final population of 77 bococizumab patients. The primary outcome was the fold change in protein levels after 12 months compared with baseline. Protein levels were compared using a paired Student *t* test or Wilcoxon signed-rank test depending on the normality of the distribution. The Benjamini-Hochberg method was used to adjust for multiple comparisons with a false discovery rate of 5%. In addition, the change in protein levels was analyzed and compared with the results from the LoDoCo2 substudy, in which the same proteomic panels were used.^1^

A total of 173 high cardiovascular risk patients were included (mean±SD age 61.7±9.8 years, and 154 [89%] male). In the 96 patients receiving placebo, there was no significant change in any of the proteins (data not shown). In the bococizumab group, mean±SD LDL-C levels were reduced from 114±41 to 47±34 mg/dL (reduction 66±29 mg/dL) while there was a neutral effect on hs-CRP levels (median change 0.0 mg/L [IQR, −0.8 to 1.1]). PCSK9 was the only protein with a statistically significant change upon treatment with bococizumab (mean±SD 24.4±11.0 versus 15.0±10.7; P_FDR_=0.033; Figure [A]). In comparison, treatment of colchicine in LoDoCo2 was associated with a significant change in 60 proteins from multiple pathways related to inflammation, the adaptive and innate immune system, neutrophil activity, and angiogenesis (Figure [B]).^1^

**Figure.**
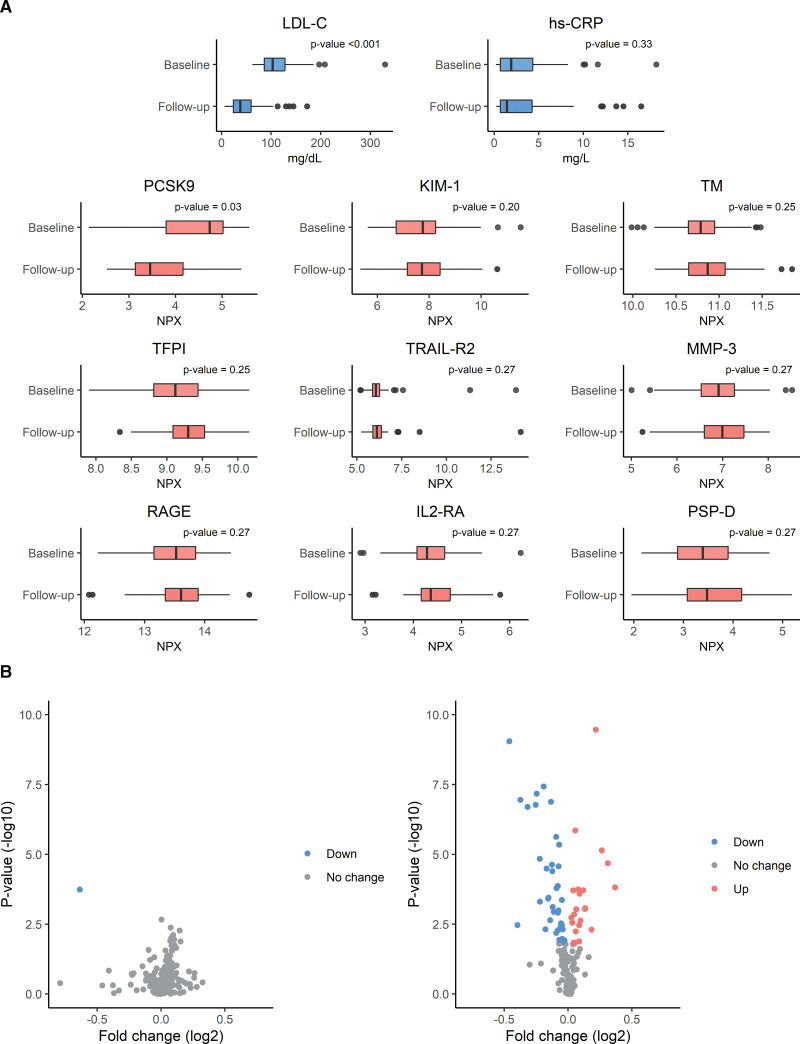
**Change of plasma proteins upon treatment with bococizumab and colchicine. A**, Boxplots of plasma proteins levels at baseline and after treatment with bococizumab of LDL-C (low-density lipoprotein), hs-CRP (high-sensitivity C-reactive protein) levels, and proximity extension assay measured proteins based on significance (P_FDR_≤0.30). **B**, Volcano plots of change (log2 fold change) in plasma protein levels upon treatment with bococizumab (left) and colchicine (right). *P* values were adjusted for multiple comparisons after which significant proteins were colored according to the direction of plasma protein change. IL2-RA indicates interleukin-2 receptor alpha chain; KIM-1, kidney injury molecule-1; MMP-3, matrix metalloproteinase-3; PCSK9, proprotein convertase subtilisin/kexin type 9; PSP-D, pulmonary surfactant-associated protein D; RAGE, receptor for advanced glycation endproducts; TFPI, tissue factor pathway inhibitor; TM, transmembrane protein; TRAIL-R2, tumor necrosis factor-related apoptosis-inducing ligand R2.

Here, we show that PCSK9 inhibition with bococizumab resulted in highly selective reduction of PCSK9 plasma levels without affecting over 30 well-known inflammatory proteins in the Olink panels, implying PCSK9 inhibition lowers atherosclerotic cardiovascular disease risk via pure lipid-lowering pathways. Our study reinforces the neutral systemic inflammatory effect of PCSK9 inhibition on top of statin therapy. The local effects on cellular and vascular inflammation by PCSK9 inhibition observed in earlier studies most likely reflect a direct consequence of a reduced cholesterol content in inflammatory cells and plaques,^2,3^ subsequently leading to a local anti-inflammatory effect. These findings support the combination of targeted anti-inflammatory therapies on top of potent LDL-C lowering to optimally address the residual inflammatory risk, as attested by recent landmark trials such as the CANTOS (Canakinumab Anti-inflammatory Thrombosis Outcome Study) and the LoDoCo2.

Our current study has several limitations. First, the sample size of this substudy was limited. Nevertheless, both changes in hs-CRP as well as LDL-C were comparable to changes in the overall SPIRE population.^4^ Second, the targeted as well as immune-based nature of the proteomic panels could have led to selection bias and potentially reduced detection of low-expressed proteins, respectively. Finally, despite the present study having a similar study population compared with LoDoCo2, the studies differed in sample size and treatment duration, which may have had impactions for direct comparison.

In conclusion, PCSK9 inhibition with bococizumab resulted in a significant reduction of circulating PCSK9 in absence of any robust change in inflammatory protein levels. The utility of implementing proteomics as a tool to unravel mechanisms for targeted therapies in high-risk patients offers promise and warrants testing in future prospective trials.

## ARTICLE INFORMATION

### Sources of Funding

This work was supported by a European Research Area Network on Cardiovascular Diseases (ERA-CVD) grant (ERA-CVD JTC2017) and the Dutch Heart Foundation (2017-20).

### Disclosures

J.H. Cornel reports membership on advisory boards for Amgen, Sanofi, Novo Nordisk en AstraZeneca. D. Gill and G.K. Hovingh are employed part-time by Novo Nordisk and own shares. A.L. Catapano reports consulting fees/lecturing fees from Akcea, Amgen, Amryt, Sanofi, Esperion, Kowa, Novartis, Ionis Pharmaceuticals, Mylan, Menarini, Merck, Recordati, Regeneron Daiichi Sankyo, Genzyme, Aegerion, and Sandoz. W. Koenig served on the Executive Steering Committee of SPIRE, which was funded by Pfizer. WK reports advisory board/lecturing fees from Novartis, The Medicines Company, DalCor, Kowa, Amgen, Corvidia, Daiichi-Sankyo, Genentech, Novo Nordisk, Esperion, OMEICOS, Sanofi, New Amsterdam Pharma, TenSixteen Bio and Bristol-Myers Squibb, grants and nonfinancial support from Abbott, Roche Diagnostics, Beckmann, and Singulex, outside the submitted work. Ridker served as the Trial Co-Chair for SPIRE, which was funded by Pfizer. Unrelated to the current project, P.M. Ridker has received additional institutional research grant support from Novartis, Amarin, Pfizer, Esperion, Novo Nordisk, and the NHLBI; has served as a consultant to Novartis, Flame, Agepha, AstraZeneca, Janssen, Civi Biopharm, Glaxo Smith Kline, SOCAR, Novo Nordisk, Uptton, Pfizer, Health Outlook, Montai Health, New Amsterdam, Boehringer-Ingelheim, Angiowave, RTI; Zomagen, Cytokinetics, Horizon Therapeutics, and Cardio Therapeutics; and receives compensation for service on the Peter Munk Advisory Board (University of Toronto), the Leducq Foundation, Paris FR, and the Baim Institute (Boston, MA). ESGS has received lecturing/advisory board fees from Amgen, Novartis, Esperion, Sanofi-Regenerion, and Akcea. NSN is co-founder of Lipid Tools. The other authors report no disclosures.
